# Transactions of the Pathological Society

**Published:** 1840-09-19

**Authors:** Edward Hallowell


### MEDICAL EXAMINER.

DEVOTED TO MEDICINE, SURGERY, AND THE COLLATERAL SCIENCES.

No. 38.] PHILADELPHIA, SATURDAY, SEPTEMBER 19, 1840. [Vol. III.

### TRANSACTIONS OF THE PATHOLOGICAL SOCIETY.

*October*, 1840.

### CASE OF CANCER OF THE STOMACH AND LIVER.

BY EDWARD HALLOWELL, M. D.

C. F. Rafinesque Smaltz, ætat. fifty-seven, had throughout life enjoyed almost uninterrupted health until the winter of 1839–40, when he was affected with constipation of the bowels, which continued until the end of the following spring. On the 15th of June he for the first time complained of nausea, with occasional vomiting and pain after eating animal food, which occurred a short time after its entrance into the stomach. Two weeks ago he observed, for the first time, a tumour in the right hypochondriac region, when he also noticed a decided yellowness of the skin; since then he has complained much of debility, and for the last week has been confined almost entirely to his room.

*Present state, Sept. 10th, 1840, when I was requested to see him in consultation by my friend Dr. Ashmead.*—Decubitus dorsal, inclined to the right side; skin throughout highly jaundiced, the conjunctiva presenting a decidedly yellow tinge; temperature of skin natural; pulse ninety-six, of moderate volume, soft and compressible; respiration twenty-eight, accelerated on exertion, or after much conversation; chest sounds well on percussion throughout; respiration pure; impulse and sounds of heart normal; tongue moist, pasty, not red at tip or edges; papillæ distinct. *Abdomen* somewhat distended; in the right hypochondriac region is a rounded tumour, hard to the touch, with the exception of a small space in the centre, which is somewhat softer than the edges; it commences a short distance below the xyphoid cartilage, extending laterally one inch to the left of the mesial line, and inferiorly to within two inches of the umbilicus; it measures five and a half inches transversely by four in length; very firm pressure upon it gives rise to pain,—but moderately made, is not productive of uneasiness; the parietes of the right side of the thorax, and a portion of the integuments of the same side of the abdomen, are infiltrated, pitting strongly on pressure; the inferior ribs and cartilages on this side appear more saillant than those of left; inferior and lateral portions of abdomen supple, not painful; urine of a deep saffron colour.

℞. Mass. ex Hydrarg. gr. ¼.

    Ext. Hyos. nig. gr. j., q. tert. hor.

Diet—sago, custards of arrow root and Iceland moss, &c.

11*th.*—12 *M.*—Has vomited once this morning; the fluid vomited is of a whitish colour, resembling water-brash; appetite greatly impaired; at a loss to know what to take that will not disagree with his stomach; bowels open once since last visit; stool rather small, of a dark colour; urine containing a reddish sabulous deposit, voided without pain; pulse ninety-four, soft and regular; respiration same as last noted; complains much of pain in the spine opposite the lower dorsal and upper lumbar vertebræ. Decubitus on left side painful from the weight of the tumour; he wishes his back bathed with a decoction of bark, which he thinks would afford him relief. He refuses to take calomel internally in any shape, even in the smallest doses.

12*th.*—Pulse rather more frequent, one hundred; skin dry; thirst; vomited twice after taking some liquid food; bowels open once since last visit; slight sensation of uneasiness after entrance of food into the stomach, but no pain.

14*th.*—Skin dry, less jaundiced, straw-coloured tint very decided; pulse more frequent, one hundred and eight; slight headach; tongue pale, and coated with a thin, whitish, and broken fur; mouth clammy; vomited yesterday morning after taking some rice broth; bowels open twice; stools dark-coloured and quite copious; has complained of some pain in the tumour, which he attributes to the drawing of an opium plaster, and also of slight pain in the bowels from gaseous distension.

℞. Infus. Quassiæ ℥ss. omn. bih.
    Ext. Hyosciam. gr. j. nocte; repetatur si non dormiat.

15*th.*—Weaker; had but little sleep bowels disturbed once; since last night has been much troubled with hiccup, which is an almost constant symptom; he attributes it to some mineral water, of which he was permitted to drink; cannot take the quassia.

℞. Morphiæ Sulphat. gr. j.
    Confect. Rosar. q. s. ft. pastill. xxiv.
Sig. Detur un. secund. hor. nocte,
    Ext. Hyosciam. gr. ss.
    Aq. ʒj.
    Tr. Hyosciam. gtt. xx. omni tert. hor.

16*th.*—Slight epistaxis, and slight vomiting of mucus tinged with blood; slept better; pulse ninety; skin dry; hiccup distressing in the afternoon of yesterday; has nearly ceased this morning; thirst great; slight headach; no discharge from the bowels; urine copious, of a deep saffron colour, highly tinged with bile.

18*th*—10 *A. M.* More feeble; evidently sinking; pulse eighty-two; respiration laboured on the slightest exertion, forty per minute; slight œdema of the feet and ankles; abdomen more distended; bowels disturbed once since last visit.

Oyster broth; weak wine and water; injections of milk.

Died at 9 P. M. The following notes of the autopsy were taken by myself and Dr. Ashmead, thirteen hours after death:

*Exterior.*—Body emaciated; straw-coloured tinge of skin; slight œdema of feet and ankles; abdomen greatly distended; no rigidity.

*Head.*—About half an ounce of serosity at base of brain; arachnoid pale and moist, of natural thickness, slightly opaque at base of brain, where it surrounds the commissure of the optic nerves; a considerable quantity of serum in subarachnoid tissue upon surface of hemispheres; none at base; cortical substance pale, from one and a half to two lines in thickness; convolutions from four lines to an inch and a half in depth; medullary substance firm, not injected; the lateral ventricles contain about ℥ss. of serosity; thalami and corpora striata of natural firmness; cerebellum of normal size, measuring four inches transversely.

*Thorax.*—Pleuræ pale; no effusion within their cavity; lungs grayish anteriorly, the left adherent to diaphragm, reddish violet posteriorly, slightly engorged at base; on cutting into them exudation of a considerable quantity of spumous serosity; beneath the pleura of lower lobe of left lung are several small bodies about a line in diameter, of a light yellow colour, and almost cartilaginous consistence; tissue of lungs crepitant, not containing any tubercles. *Pericardium* pale, about ℥ij. of yellowish serum within its cavity. *Heart* of normal size; left ventricle six lines in thickness, right one and a half; a few atheromatous deposits beneath lining membrane of aorta at its origin; endocardium healthy.

*Abdomen.*—About half an inch of fat beneath the integuments; peritoneum pale and moist, containing about half a gallon of lemon-coloured serum; liver greatly enlarged, its anterior margin extending three inches below the margin of the ribs, forming the tumour which was felt during life. The following adhesions are noticed connecting it with the surrounding viscera: first, adhesion of omentum to under surface of left lobe; second, adhesion of transverse colon at its commencement on right side to inferior surface of right lobe, immediately to right of gall-bladder; third, strong adhesions of upper surface of left lobe to diaphragm along its anterior margin in a space one inch in extent. The omentum is also adherent to the peritoneum at the lower edge of the right ribs. The following are the admeasurements of the liver when removed from the body: length of longitudinal diameter of right lobe, measured in a straight line, ten inches; transverse, eight inches; transverse of left, four inches; length of left lobe along suspensory ligament, five inches; circumference of liver, thirty-four and a quarter inches; its superior surface is of a reddish chocolate colour, presenting numerous elevations, the largest of which is four inches in length, by five in breadth; the whole surface of the liver is studded with a number of irregularly rounded spots of a whitish yellow colour, firm and cartilaginous to the touch; the larger ones present numerous small vessels showing a disposition to radiate upon their surface; the injection for the most part appears to be confined to the subperitoneal cellular tissue, and does not extend to the tumours themselves. The inferior aspect of the liver presents the same general appearance as the upper, but is more bosselated; on the under surface of the left lobe is a large protuberance, measuring twelve inches in circumference; it is for the most part occupied by the yellowish white matter above noticed, which is deposited in masses throughout its substance; under surface of right lobe very irregular from the presence of numerous whitish deposits, the largest of which is about an inch and a half in diameter; the surface of these tumours is greatly injected, and more or less ecchymosed; lobulus quartus greatly enlarged, presenting nearly the same general appearance as the rest of the under surface of this lobe; on cutting into the left lobe, the natural tissue of the liver appears to be obliterated, with the exception of a few points along its anterior margin; the greater part of this portion of the liver is occupied by an immense mass of altered tissue of a light yellow colour, cutting firmly under the knife, but which, when scraped by it, yields a considerable quantity of yellowish matter of almost cheesy consistence, leaving behind it a firmer portion, which appears to consist of vessels and cellular substance; numerous vessels are distributed throughout other portions of this lobe, giving it a high degree of vascularity, and a considerable quantity of blood is extravasated through it; those portions of the liver which appear least altered are much softened; on cutting into the right lobe the following appearances are presented: general colour greenish yellow, the two substances being distinct and moderately firm; at the point of junction of the two surfaces anteriorly is a deposit of a light pink colour, one inch and a half in diameter, very much softened, (medullary sarcoma;) the rest of this lobe is filled with numerous masses of a light yellow colour, more or less vascular; minute radiating vessels are seen in several of them, and others have a considerable quantity of blood extravasated throughout their substance; the radii appear to extend from the centre towards the circumference; gall-bladder moderately distended with bile, projecting about one inch below anterior margin of liver; length six inches; it contains a quantity of thin bile of a light yellow colour; in it are found seventy-two calculi of various sizes, and irregular in shape, all of them angular, with smooth surfaces, the largest about the size of a small filbert. *Stomach* greatly distended, pale externally; it contains a quantity of fluid of the consistence of gruel, without perceptible odour; within an inch of the cardiac orifice, along the lesser curvature, is a large tumour, about three inches in diameter, projecting half an inch or more beyond the surrounding surface; it is of a cauliflower shape, presenting numerous irregular pendulous masses upon its inner aspect of soft brain-like texture, with a considerable quantity of blood extravasated through it; the surface only of the fungus presents this softened consistence; internally its texture is firm and almost cartilaginous, containing a quantity of yellowish matter, which can be squeezed from it in points; the mucous membrane of the anterior and posterior faces of the stomach is nearly destroyed; elsewhere it is of a pale onion tint, except in the immediate neighbourhood of the tumour, where it is somewhat injected and thinner than natural, adhering strongly to the subjacent coat. *Pancreas* adherent to the external coat of the stomach opposite the tumour; that portion of it immediately in contact with the stomach is altered in its texture, presenting the usual appearances of scirrhus; the other portions are healthy; intestines pale externally, much distended with gas; mucous membrane examined in points pale, and of normal consistence; caput coli healthy; no disease of mesenteric glands. *Spleen* five inches in length, by three in breadth, presenting its usual appearance and consistence. *Left kidney* four inches and three quarters in length by two and a half in breadth; thickening and opacity of its proper tunic anteriorly from old inflammation; cortical substance three lines in thickness, of an obscure yellow colour, presenting numerous reddish streaks; in the anterior and inferior portion of the right kidney is a cyst large enough to contain a filbert, filled with urine; it is in contact with the proper membrane of the kidney anteriorly. *Bladder* moderately distended, containing a quantity of deep saffron-coloured urine; lining membrane healthy; prostate not enlarged.

*Observations.*—The above case furnishes a well-marked example of cancer of the stomach and liver. The two are not unfrequently combined, and the diagnosis is sometimes a matter of difficulty. Cancer of the stomach, according to Andral, cannot be diagnosticated with precision unless a tumour be present in the epigastrium, for the other symptoms may be produced by chronic inflammation; there are, however, two signs, which, conjoined, are sufficient to induce a strong suspicion of the existence of a cancerous affection, viz. the straw-coloured tint of the skin, and marked emaciation; if to these general signs be added those of a local nature, dependent upon the altered form and disturbed functions of the organ, we shall in a large proportion of cases be enabled to detect the existence of the disease in question.

The most important sign of cancer of the liver, according to M. Cruveilhier, is the sensation of tumours projecting more or less upon its surface; the other symptoms, as pain on pressure, emaciation, &c., are of little value. When the liver is greatly enlarged, as in the case of Mr. Rafinesque, the diagnosis is rendered more easy; but instances do occur where it is nearly impossible to form a correct opinion upon the subject,—as for instance when the organ is not increased in size, and when, as is frequently the case, there is no pain or other appreciable symptom; it occasionally happens also that the right extremity of the stomach, enormously enlarged, adheres to the under portion of the liver, forming with it a continuous surface, in which case the liver may be supposed to be cancerous when no trace of the disease exists.

